# The Therapeutic Potential of Baicalin and Baicalein in Breast Cancer: A Systematic Review of Mechanisms and Efficacy

**DOI:** 10.3390/cimb47030181

**Published:** 2025-03-11

**Authors:** Bartłomiej Zieniuk, Şuheda Uğur

**Affiliations:** Department of Chemistry, Institute of Food Sciences, Warsaw University of Life Sciences, Nowoursynowska 159c, 02-776 Warsaw, Poland; suheda_ugur@sggw.edu.pl

**Keywords:** breast cancer, baicalin, baicalein, flavonoids, anticancer mechanisms, apoptosis, nanotechnology

## Abstract

Cancer remains a leading cause of death globally, with breast cancer being the most commonly diagnosed cancer in women. This systematic review focuses on the therapeutic potential of baicalin and baicalein, two bioactive flavonoids derived from *Scutellaria baicalensis*, in breast cancer treatment. These compounds exhibit anticancer properties through mechanisms such as apoptosis induction, cell cycle arrest, and inhibition of metastasis. Baicalin and baicalein modulate key signaling pathways, including NF-κB, PI3K/AKT/mTOR, and Wnt/β-catenin, and have shown efficacy in both in vitro and in vivo models. Their synergy with chemotherapy agents and incorporation into nanotechnology-based delivery systems highlight opportunities to enhance therapeutic outcomes. However, current evidence is predominantly preclinical, with limited clinical trials to validate their safety and efficacy in humans. Challenges such as poor bioavailability and rapid metabolism also underscore the need for advanced formulation strategies. This review synthesizes current evidence on the molecular mechanisms, therapeutic efficacy, and potential applications of baicalin and baicalein in breast cancer research.

## 1. Introduction

Cancer is currently one of the most significant public health problems worldwide and ranks high among causes of death. According to the 2018 GLOBOCAN report, which examined global cancer incidence (the number of new cases) and death rates across 185 countries for 36 types of cancer, it was estimated that there were approximately 18.1 million new cancer cases and 9.6 million cancer-related deaths globally [1]. Lung cancer, the most prevalent type, accounted for 11.6% of total cases and 18.4% of cancer deaths. When examined in terms of death, the most striking rates were colorectal cancer (9.2%), followed by stomach cancer (8.2%) and liver cancer (8.2%). Lung cancer remains the most common and deadly type among men, while breast cancer is the most frequently diagnosed in women. Furthermore, in developed countries, the most common cancers are lung, breast, and prostate cancers. In contrast, in low-income countries, there is a higher prevalence of infection-related cancers such as liver and cervical cancers [1].

According to the 2020 GLOBOCAN data, there were 19.3 million new cancer cases and 10 million cancer-related deaths worldwide. In 2020, for the first time, breast cancer surpassed lung cancer to become the most commonly diagnosed type globally [2]. The most recent data from the 2022 GLOBOCAN report indicate that lung cancer remains the most frequently diagnosed and deadliest type of cancer worldwide, with 2.48 million cases constituting 12.4% of all cancers [3]. In women, breast cancer is the most commonly diagnosed type, with an increasing trend of approximately 2.3 million new cases annually [4]. Breast cancer, with 25% of all female cancers, represents 15% of female cancer deaths [3]. Risk factors for breast cancer include age, physical inactivity, smoking and alcohol consumption, and obesity.

Breast cancer types are classified based on hormone receptors and human epidermal growth factor receptor 2 (HER2+). Estrogen receptor-positive breast cancer is the most frequently occurring subtype [4]. Endocrine therapies help to reduce the effects of estrogen that promote tumor growth for Hormone Receptor Positive (HR+) and ERBB2-Negative types. The treatment duration typically depends on regular oral medication intake over five to ten years. In cases where hormone therapy is not enough, chemotherapy may also be administered to patients exhibiting high resistance to treatment. For ERBB2-Positive (HER2/ERBB2+) cancers, treatments targeting HER2 protein (such as Trastuzumab and Pertuzumab) are combined with chemotherapy. Triple-negative Breast Cancer (TNBC) is treated with chemotherapy as well. Various new therapeutic approaches are being developed for breast cancer treatment, including PD-1 inhibitors like Pembrolizumab and Atezolizumab as alternative treatment options [5]. In HR+ breast cancer, CDK4/6 inhibitors help regulate the cell cycle and slow down disease progression when combined with chemotherapy [6]. In hormone receptor-positive breast cancers, Tamoxifen and Aromatase inhibitors support treatment by preventing estrogen from promoting tumor growth [6]. In early-stage breast cancer cases, various treatment methods such as Breast-Conserving Therapy (BCT) and Intraoperative Radiotherapy (IORT) are also observed [7]. While advances in targeted therapies and immunotherapies have improved survival rates, significant challenges persist. For instance, the initial chemotherapy response is about 40% in patients with triple-negative breast cancer. Still, at relapse, most have metastatic disease, primarily in the brain, bones, and lungs, resulting in a poor 5-year survival rate of roughly 12% [8]. Endocrine therapies often fail in metastatic hormone receptor-positive (HR+) cases due to acquired resistance [9]. Additionally, severe side effects—such as cardiotoxicity from trastuzumab [10] and hematologic complications from chemotherapy [11] limit treatment adherence and efficacy. These limitations underscore the urgent need for safer, multitargeted agents to circumvent resistance mechanisms and minimize adverse effects.

Natural polyphenols, such as flavonoids, have gained attention for their anticancer properties. Polyphenols can support cancer treatment by reducing cell proliferation, promoting apoptosis, suppressing inflammatory responses, and preventing tumor angiogenesis. Studies on polyphenols’ effects on specific types of cancers, such as melanoma, colorectal cancer, and lung cancer, indicate that they can avoid metastasis in melanoma and lung cancers while exhibiting synergistic effects when combined with chemotherapy in colorectal cancer treatments [12].

Stilbenes, like resveratrol, can inhibit tumor cell growth and reduce inflammation [13]. Phenolic acids exhibit antioxidant activity that can prevent DNA damage. Curcumin, a phenolic compound with anti-inflammatory and antioxidant properties, has shown positive results in colon cancer treatment [14]. Flavonoids can regulate cell signaling pathways and enhance tumor cell sensitivity while exhibiting effects similar to estrogen in breast cancers to inhibit the proliferation of cancer cells [15]. Resveratrol also prevents DNA damage and supports tumor cell death [16]. As seen in many examples, polyphenols were used as an adjuvant or a natural treatment method for cancer due to their antioxidant nature.

**Figure 1:**
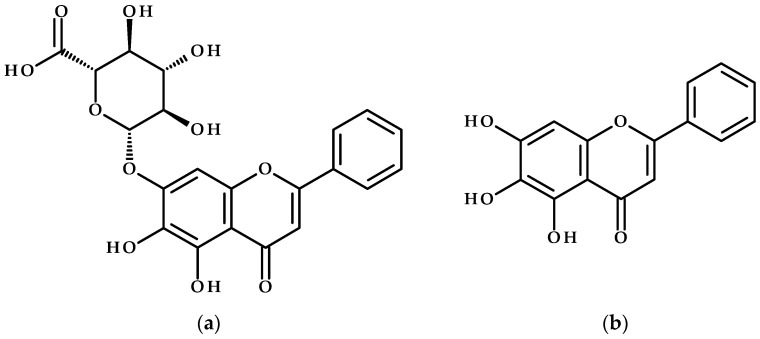
Chemical structures of (**a**) baicalin and (**b**) baicalein.

Baicalin and baicalein (Figure 1) are two prominent bioactive flavonoids widely recognized for their diverse pharmacological properties, including anti-inflammatory, antioxidant, antiviral, and anticancer activities [17]. These compounds are primarily derived from the roots of *S. baicalensis*, a plant extensively used in traditional Chinese medicine for centuries to treat conditions such as fever, cough, and infections [18]. While *S. baicalensis* is the most well-known source of baicalin and baicalein, these flavonoids can also be found in other plants but in smaller quantities. For example, other *Scutellaria* species, *Phyllanthus amarus*, *Vernonia amygdalina*, *Tuber aestivum*, *Xanthium spinosum*, or *Centaurea mersinensis* [19,20,21,22,23,24].

Baicalin has poor oral bioavailability due to low water solubility and limited membrane permeability. In contrast, its aglycone metabolite, baicalein, formed by removing glucuronic acid, exhibits superior lipophilicity and permeability for efficient absorption. Baicalin is hydrolyzed to baicalein during intestinal absorption by gut microbiota and enzymes. Conversely, UDP-glucuronosyltransferase enzymes in enterocytes and the liver can reconvert baicalein back to baicalin. This metabolic interplay enhances the compound’s pharmacodynamic profile: while baicalin is poorly absorbed, conversion to baicalein facilitates systemic uptake, with re-glucuronidation potentially prolonging therapeutic effects [25]. Notably, baicalein has shown dual antioxidant/pro-oxidant activity, inducing oxidative stress in cancer cells without harming healthy tissues [26]. Mechanistically, they selectively target pathways dysregulated in breast cancer, such as PI3K/AKT/mTOR and STAT3, while sparing normal cells, a critical advantage over conventional chemotherapies [27,28].

This systematic review aims to comprehensively evaluate the roles of baicalin and baicalein in breast cancer by synthesizing current scientific evidence on their mechanisms of action, therapeutic efficacy, and potential as adjunctive or standalone treatments. Specifically, it seeks to elucidate their molecular mechanisms in modulating key pathways such as apoptosis, autophagy, oxidative stress, and inflammation and assess their anticancer effects, including tumor growth inhibition and metastasis suppression. This review aims to highlight the potential of baicalin and baicalein as promising therapeutic agents in breast cancer treatment by providing a critical and up-to-date analysis.

## 2. Materials and Methods

The first methodological tool used in the current study was bibliographic analysis. Data were collected from the Scopus database, which was accessed on 1 February 2025. The search query used was TITLE-ABS-KEY (“baicalin” OR “baicalein”). CSV file was exported from the Scopus database and imported into a Microsoft Excel 2021 (Microsoft Corporation, WA, Redmond, USA) spreadsheet for additional analysis. Initially, an examination of document types, languages, journals, countries, and the number of publications over the years was conducted.

Following this, data from all selected publications (totaling 10,278) were analyzed using VOSviewer version 1.6.20 (Centre for Science and Technology Studies, Leiden University, Leiden, The Netherlands). The author’s keywords were analyzed using co-occurrence analysis, with the minimum number of occurrences set to 75. This process yielded a total of 39 distinct keywords.

A systematic review was conducted following the Preferred Reporting Items for Systematic Reviews and Meta-Analyses (PRISMA) guidelines [29]. The completed PRISMA checklist is provided as Table S1 in the Supplementary Materials. A systematic literature search was performed in the Scopus database, but the following keyword combination was employed to ensure a comprehensive retrieval of relevant studies: (TITLE-ABS-KEY (“baicalin” OR “baicalein”) AND TITLE-ABS-KEY (breast) AND TITLE-ABS-KEY (“cancer” OR “tumor” OR “carcinoma” OR “malignancy”)). For inclusion criteria, the studies were eligible if they: (a) examined the effects of baicalin or baicalein on breast cancer (in vitro, in vivo, or clinical studies), (b) were original research articles (e.g., experimental studies, preclinical trials), (c) provided full-text access, and (d) reported outcomes related to mechanisms, efficacy, or therapeutic applications. In the case of exclusion criteria, the studies were excluded if they: (a) were not published in English, (b) lacked full-text availability (e.g., conference abstracts, letters without data), (c) were non-original research (e.g., reviews, editorials, commentaries), and (d) did not directly investigate breast cancer or baicalin/baicalein.

Two authors independently screened all retrieved records in two stages: (a) title/abstract screening, i.e., irrelevant studies, non-English papers, and non-original articles were excluded, and (b) full-text review, i.e., remaining studies were assessed against inclusion/exclusion criteria. Discrepancies between the two reviewers were resolved through discussion until a consensus was reached.

## 3. Results

### 3.1. Bibliometric Analysis of Publications on Baicalin and Baicalein

**Figure 2:**
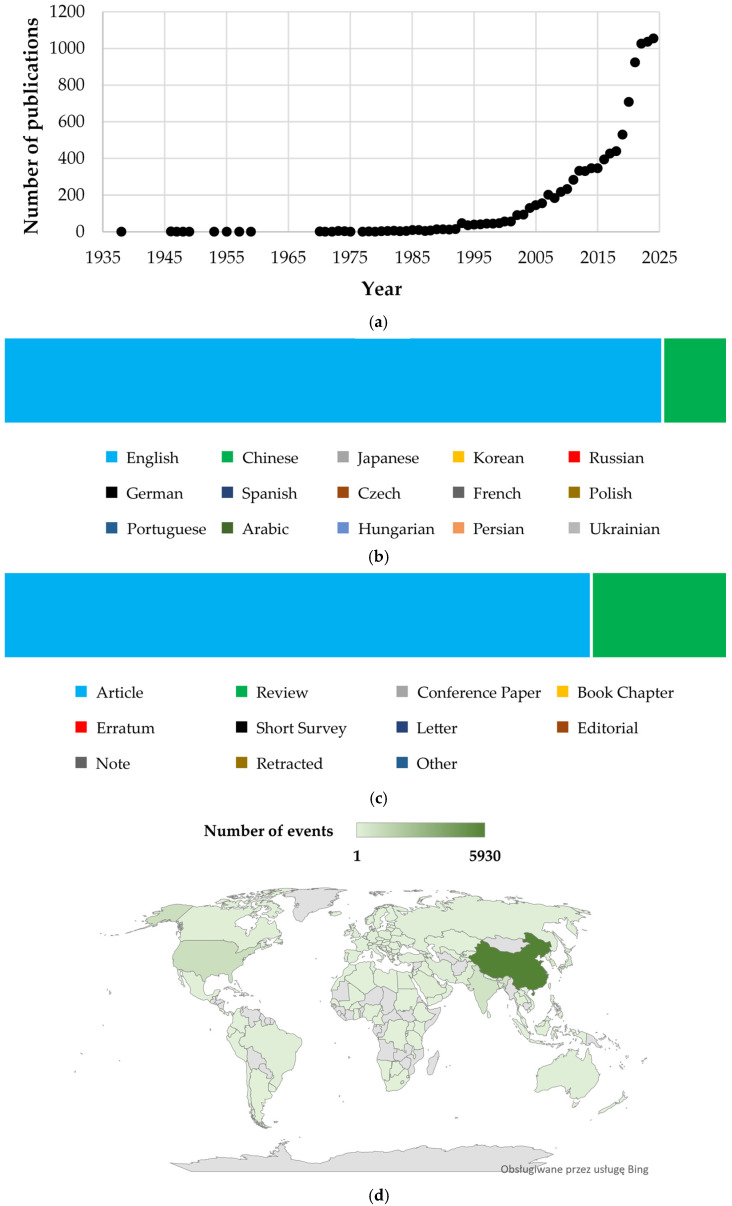
Visualization of bibliometric analysis of baicalin and baicalein-related research: (**a**) annual publication trend, (**b**) distribution of papers by language, (**c**) classification of papers by type, and (**d**) global contributor map.

The annual publication trends for baicalin and baicalein reveal a surge in research interest, particularly over the past decade (Figure 2a). After minimal output in the 20th century, publications grew steadily from 16 in 1992 to 57 in 2000, then exponentially to 709 in 2020. The early 2020s saw unprecedented growth, with annual publications exceeding 1000 papers (2022–2024), reflecting a heightened focus on their therapeutic potential in addressing global health challenges, such as the COVID-19 pandemic [30], where baicalin and baicalein were studied for their potential antiviral effects. Additionally, their applications in cancer therapy, neuroprotection, and metabolic disorders have garnered significant attention [27].

The distribution of scientific documents on baicalin and baicalein by language (Figure 2b) highlights the global reach of research on these compounds. The vast majority of publications (*n* = 8929; 86.9%) are in English, the universal language of scientific research. Chinese is the second most common language, with 1253 publications (12.2%). This aligns with the origins of baicalin and baicalein in traditional Chinese medicine and the significant contributions of Chinese researchers to this field. The presence of multiple languages (Japanese, Korean, Russian, etc.) underscores the worldwide interest in the research on baicalin and baicalein.

Figure 2c illustrates the distribution of scientific documents concerning baicalin and baicalein by document type. Original research papers, which include articles, make up the majority of these publications, totaling 7931 documents (77.2%). Reviews follow as the second most frequent type, comprising 1972 publications (19.2%).

Research on baicalin and baicalein spans over 100 countries. A world map (Figure 2d) visualizes the global distribution of research contributions on these topics, highlighting China as the dominant hub of activity. Significant research clusters also emerge across the Americas (United States, Brazil), Europe (United Kingdom, Italy, Germany), Asia (India, Japan, South Korea), and parts of Africa (Egypt, Nigeria) and the Middle East (Saudi Arabia, Iran).

**Figure 3:**
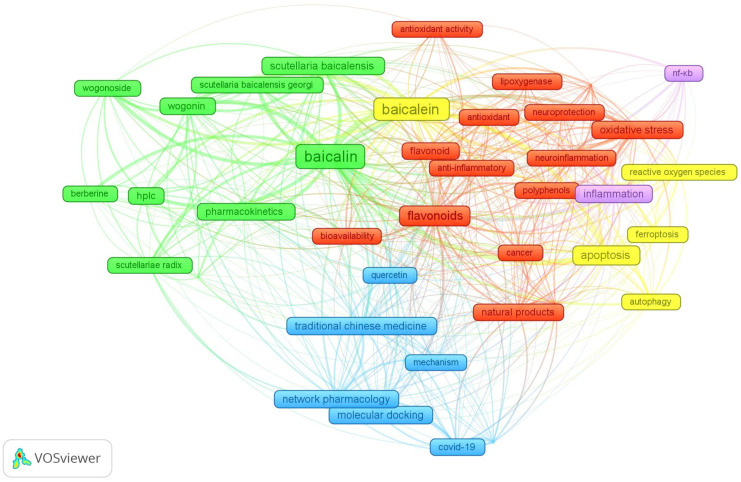
Keyword co-occurrence network visualization generated using VOSviewer.

The cluster analysis of authors’ keywords (*n* = 39) on baicalin and baicalein reveals five distinct clusters (Figure 3), each focusing on specific aspects of these compounds’ pharmacological properties, mechanisms, and applications.

Cluster 1 (red) highlights the broad pharmacological effects of baicalin and baicalein, including anti-inflammatory, antioxidant, and neuroprotective properties, and their potential in cancer therapy as bioactive flavonoids and polyphenols. Cluster 2 (green) focuses on phytochemistry and analytical studies, including extraction, quantification (HPLC), quality control, pharmacokinetics, and related compounds like wogonin and wogonoside. Cluster 3 (blue) emphasizes their application in COVID-19 through molecular docking and network pharmacology, alongside their significance in traditional Chinese medicine and potential synergy with other compounds. Cluster 4 (yellow) explores their impact on cell death pathways (apoptosis, autophagy, ferroptosis) and modulation of reactive oxygen species (ROS) linked to diseases like cancer. Lastly, Cluster 5 (violet) centers on their anti-inflammatory effects and modulation of the NF-κB signaling pathway. Together, these clusters showcase the versatile research landscape of baicalin and baicalein and their therapeutic potential in modern and traditional medicine, addressing global health issues like cancer and COVID-19.

Furthermore, Table 1 below is a summary of the five most frequently cited scientific publications in the Scopus database that reference baicalin and baicalein. These publications cover various aspects of polyphenols’ pharmacological properties, including antimicrobial activity, health benefits, and therapeutic applications. Cushnie and Lamb [31] lead with 3287 citations, reflecting their foundational work on the antimicrobial properties of flavonoids, including baicalin and baicalein. Del Rio et al. [32] follow with 2067 citations, emphasizing the health benefits of dietary polyphenols and their role in chronic disease prevention. Wu et al. [33], with 1712 citations, gained rapid recognition for their focus on COVID-19 therapeutic targets and the potential of baicalin, baicalein, and other bioactive compounds as antiviral agents. Moreover, this paper is the only original research paper in this set. Formica and Regelson [34], a classic review on quercetin and related bioflavonoids, has 1617 citations. Finally, Zhang et al. [35], with 1331 citations, provide a comprehensive review of extraction and isolation techniques for natural products, highlighting their importance in natural product research. The papers mentioned above demonstrate the diverse applications of polyphenol compounds, from antimicrobial and antiviral activities to chronic disease prevention and methodological advancements, and their high citation counts reflect the global scientific community’s interest in the therapeutic potential of these bioactive compounds.

### 3.2. Characteristics of Included Studies

**Figure 4:**
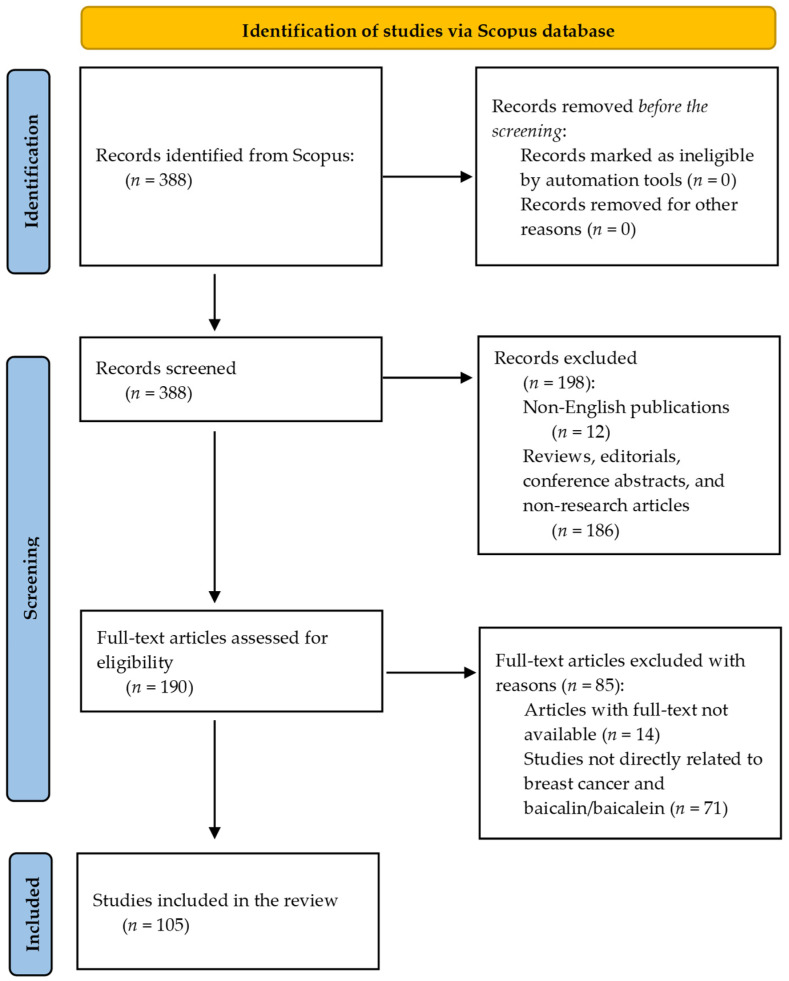
The PRISMA flowchart for the selection and screening of the articles.

In the case of systematic review, the initial search yielded 388 papers published up to 1 February 2025. After applying language filters, 376 articles were retained, as they were published in English. Subsequently, the articles were screened based on their document type, identifying 190 original research articles. Following a revision process to ensure relevance and eliminate articles with full text not available and studies not directly related to breast cancer and the application of baicalin or baicalein, 105 articles were deemed eligible for inclusion in this review. In the exclusion criteria, 12 papers were excluded for being non-English, 186 for not being original research articles, 14 due to lack of full-text availability, and 71 because they were not directly related to breast cancer and the application of baicalin/baicalein. The PRISMA flowchart is shown in Figure 4.

The 105 articles were classified into six thematic categories according to their primary focus on (a) baicalin, (b) baicalein, (c) plant extracts and herbal prescription used in traditional medicine, (d) derivatives of baicalein, (e) combination therapy, and (f) nanoparticle-based delivery systems.

### 3.3. The Use of Baicalin in the Treatment of Breast Cancer

Baicalin, a flavonoid glycoside, and glucuronide of baicalein, has demonstrated significant anticancer properties in various breast cancer models. Table 2 provides a summary of the research studies concerning the role of baicalin in combating breast cancer [35,36,37,38,39,40,41,42,43,44,45,46,47,48,49,50]. To date, 16 papers have been published on this topic, with the first appearing in 2001. Interestingly, Ikezoe et al. [36] demonstrated that baicalin could not inhibit the growth of the MCF-7 cell line. In other studies, the result was quite the opposite.

Baicalin has been shown to inhibit the proliferation of various breast cancer cell lines. Both Wang et al. [37] and Ge et al. [46] reported that baicalin inhibited MCF-7 cell proliferation in a dose- and time-dependent manner. At 200 µM, baicalin induced strong cytotoxicity and morphological changes indicative of cell death, arresting cells in the G0/G1 phase and upregulating p53 and the bax gene [37]. Moreover, with miRNA microarray analysis, Ge et al. [46] identified 92 upregulated and 35 downregulated miRNAs at 150 µM of baicalin.

Furthermore, baicalin induced apoptosis in different breast cancer cell lines through multiple mechanisms. It was evidenced by, e.g., chromatin condensation and upregulation of p53 and bax [37], increasing cytochrome c release, DNA fragmentation, and caspase-3, -8, and -9 activity in MDA-MB-231 and MCF-7 cells [45], as well as, by heightened ROS production in MCF-7 cells [50].

Anti-metastatic effects in breast cancer cell lines were also observed. In the vast majority of studies, baicalin inhibits breast cancer metastasis by targeting migration, invasion, and EMT, upregulating E-cadherin, and downregulating mesenchymal markers like Vimentin and N-cadherin [40,42,49].

In addition, signaling pathways involved in cancer progression were modulated by baicalin. Chung et al. [38] reported that baicalin inhibited NF-κB activation and TGF-β1-induced EMT in MCF-10A and MDA-MB-231 cells, suppressing migration and colony formation. Gao et al. [41] found that baicalin inhibited breast cancer cell proliferation, invasion, and migration by inducing G1/S arrest and suppressing the NF-κB pathway. Chen et al. [47] demonstrated that baicalin suppressed luminal breast cancer (ZR-75-1) growth by downregulating ESR1 but promoted triple-negative breast cancer (MDA-MB-231) growth by upregulating PROCR.

Finally, several in vivo studies have demonstrated the therapeutic efficacy of baicalin. Gao et al. [41] reported that baicalin reduced tumor growth in BALB/c nude mice and modulated inflammatory responses. Liu et al. [44] found that baicalin suppressed tumor growth and metastasis in BALB/c-null mice, upregulating E-cadherin and downregulating TGF-β1, vimentin, and p-Smad3 in tumors. Wang et al. [48] demonstrated that baicalin reversed bone loss in bone cancer pain rats, inhibiting osteoclast activation and reducing inflammation. Jia et al. [51] reported that baicalin suppressed epinephrine-induced migration and invasion of breast cancer cells, protecting against chronic stress-promoted metastasis in BALB/c and C57BL/6J mice.

### 3.4. Studies Investigating Baicalein as an Anticancer Agent

Baicalein, the aglycone of baicalin, was more commonly researched [26,52,53,54,55,56,57,58,59,60,61,62,63,64,65,66,67,68,69,70,71,72,73,74,75,76,77,78,79,80,81,82,83,84,85,86,87,88,89,90,91] (Table 3). Similarly to baicalin, baicalein has been shown to inhibit the proliferation of various breast cancer cell lines. The study of So et al. [52] was the first to demonstrate baicalein’s ability to inhibit MDA-MB-435 cell proliferation. Baicalein consistently inhibited the proliferation of breast cancer cell lines such as MDA-MB-435, MCF-7, MDA-MB-231, and 4T1. So et al. [52,53] reported that baicalein inhibited the proliferation of MDA-MB-435 and MCF-7 cells, with IC_50_ values of 5.9 µg/mL and 5.3 µg/mL, respectively. Interestingly, Kuntz et al. [54] found that MCF-7 cells were less sensitive to baicalein than intestinal cells. Ragazzon et al. [62] demonstrated that baicalein-induced G1-phase arrest in MCF-7 cells and caused DNA damage, highlighting its antiproliferative effects.

Mitochondrial dysfunction and caspase activation [55,60], modulation of Bcl-2 family proteins [55], and increased intracellular Ca^2+^ levels and ROS production [60,70] were observed as the response for baicalein-induced apoptosis.

Moreover, baicalein demonstrated potent anti-metastatic properties by inhibiting cancer cell adhesion, migration, and invasion. It suppressed the activity and expression of matrix metalloproteinases (MMPs), such as MMP-2 and MMP-9, which are critical for cancer cell invasion [64]. Baicalein also reversed epithelial-mesenchymal transition (EMT), a key process in metastasis, where Chen et al. [82] demonstrated that baicalein delayed tumor progression, reduced metastasis, and prolonged survival in a breast cancer mouse model by suppressing calpain-2 activity and modulating Ca^2+^ levels and ERK signaling. In another study, Gao et al. [71] found that baicalein downregulated SATB1 protein expression, inhibiting the proliferation and migration of MDA-MB-231 cells.

Baicalein targeted multiple signaling pathways, including MAPK/ERK/p38 pathway [63], PI3K/AKT/mTOR pathway [78], Wnt/β-catenin pathway [73], STAT3 signaling [84], and KDM4E/BICD1/PAR1 [88].

Regarding in vivo studies, in animal models, such as Sprague-Dawley rats, BALB/c mice, and SCID mice [69,74,88], baicalein significantly suppressed tumor growth, reduced metastasis, and prolonged survival. In detail, Wang et al. [69] reported that baicalein treatment significantly suppressed tumor growth without toxicity in an orthotopic mouse model of triple-negative breast cancer.

Baicalein exhibits potent anticancer properties in breast cancer models, including antiproliferative, pro-apoptotic, and antimetastatic effects. The compound modulates multiple signaling pathways and is efficacious in combination therapies and in targeting cancer stem cells. Baicalein showed limited effectiveness against breast cancer stem cells (CSCs) compared to other natural compounds like ginsenoside F2, tangeretin, and nobiletin [66].

Additionally, numerous recent studies have employed techniques like network pharmacology, molecular docking, and molecular dynamics simulations to minimize costs and accelerate drug discovery. As one example of such studies, He et al. [92] investigated the compound banmao capsule, a widely used antitumor mixture consisting of 11 herbs. The authors identified 128 compounds and 436 targets of this traditional medicine remedy, and moreover, VEGF (Vascular Endothelial Growth Factor) and estrogen signaling pathways seem to play a role in its antitumor activity. Similar studies, e.g., Qi et al. [93], aimed to investigate the anticancer mechanism of *S. barbata*, where the authors identified the active constituents of this plant, predicted their targets, and analyzed their interactions. Another study examined the potential of baicalein as an inhibitor of cyclooxygenase-2 (COX-2), a key enzyme linked to breast and ovarian cancers [94]. Finally, the study of Rathi et al. [95] explored the potential of baicalin and resveratrol as inhibitors of PIM-1 kinase, which plays a critical role in cancer progression by regulating cell proliferation, survival, and signaling pathways like PI3K/Akt/mTOR and JAK/STAT.

### 3.5. Anticancer Effects of Plant Extracts Containing Baicalein and Baicalin in Breast Cancer Models

As mentioned earlier, *S. baicalensis* is the primary source of baicalin and baicalein, but these compounds also occur in plants in other regions of the world. *S. baicalensis* has been utilized as a medicine in various East Asian countries for over 2000 years [18]. The studies included in the systematic review focused on extracts from *Scutellaria* species, *P. amarus*, *T. aestivum*, *V. amygdalina*, *X. spinosum*, and *C. mersinensis*, as well as Sanhuang Xiexin Decoction (SXD) and Huangqin Tang (HQT) formulations [19,20,21,22,23,24,96,97,98,99,100,101,102]. These extracts exhibited different levels of baicalein and baicalin, which influenced their distinct anticancer properties, and the data are summarized in Table 4.

Parajuli et al. [19] demonstrated the anticancer potential of thirteen Scutellaria species extracts in MDA-MB-231 cells, i.e., *S. alpina*, *S. angulosa*, *S. baicalensis*, *S. barbata*, *S. costaricana*, *S. integrifolia*, *S. lateriflora*, *S. montana*, *S. ocmulgee*, *S. ovata*, *S. racemosa*, *S. scandens*, and *S. suffrutescens*. The extracts contained varying levels of six flavonoids, with baicalin being the most abundant in most species. The extracts contained baicalein (0.21–2.34 μg/mg) and baicalin (0.92–28.16 μg/mg), with the highest values observed for the leaves of *S. angulosa*. The flavonoids showed potent antiproliferative and proapoptotic activities, with mechanisms involving cell cycle arrest (G1/G2 phase) and induction of apoptosis [19]. The extracts from *S. baicalensis* were also studied by Wang et al. [96], Yu et al. [97], and Park et al. [98]. Wang et al. [96] compared different fractions of the extracts with varied contents of baicalin and baicalein, and they found that a baicalin-deprived fraction, primarily containing baicalein and wogonin, demonstrated the most potent anticancer activity in MCF-7 cells and effectively induces apoptosis in MCF-7 cells. In contrast, other fractions did not exhibit similar effects, and cell cycle arrest was also observed. Yu et al. [97] reported that cellulase pretreatment was applied to enhance the antiproliferative potential of *S. baicalensis* extracts against MCF-7 and HCT-116 cells. The authors first optimized the deglycosylation conditions and found that 20 U/g cellulase at pH 4.8 and 50 °C for 8 h, leading to obtaining promising bioactive extracts. The final extract induced S-phase cell cycle arrest in HCT-116 cells and significantly increased early and late apoptosis rates in a dose-dependent manner [97].

Moreover, the aqueous extract of *P. amarus* mitigated Cr(VI)-induced oxidative toxicity in MDA-MB-435S cells [20]. Beara et al. [21] found baicalein in extracts of *T. aestivum* (black truffles), which notably hindered growth in both MCF-7 and HeLa cells. In contrast, *T. magnatum* (white truffles) lacked both baicalin and baicalein. The ethyl acetate extract of *V. amygdalina* leaves exhibited anticancer activity against 4T1 breast cancer cells by inducing apoptosis, enhancing cell accumulation in the G2/M phases of the cell cycle, and inhibiting the expression of PI3K and mTOR [22]. *X. spinosum* was another species that contained baicalin or baicalein either in methanol or hexane fractions [23]. Treatment with *X. spinosum* extracts significantly reduced tumor size in BALB/c mice, particularly with aqueous, methanol, and chloroform fractions. Moreover, the aqueous fraction significantly increased caspase-3 activity, suggesting a strong induction of apoptosis in cancer cells. In addition, no toxic effects on liver or kidney functions were observed in treated mice [23]. Finally, Yırtıcı et al. [24] investigated the bioactive properties of *C. mersinensis*, an endemic species in Turkey. The methanol extract contained significant phytochemicals, including scutellarin, chlorogenic acid, and baicalin. In vitro studies demonstrated that the methanol extract exhibited cytotoxic effects against breast cancer cells (MCF-7), which was more effective than against other breast cancer cell lines (MDA-MB-231 and SKBR-3) with IC_50_ values ranging from 22.17 to 46.20 µg/mL.

Traditional remedies and medicines, renowned for their extensive historical use, have also been evaluated for their potential as anticancer agents. Du et al. [99] assessed the Shuganning injection (SGNI). SGNI is a reformulation of the traditional decoction, first documented 1800 years ago. It consists of four herbal extracts: *Ganoderma lucidum*, *Isatidis radix*, *Gardeniae fructus*, and *Artemisiae scopariae*, along with baicalin. SGNI was approved by the China Food and Drug Administration (CFDA) as a traditional Chinese patent medicine in 2002 and is used to treat conditions such as hepatitis, high bilirubin levels, liver damage, fatty liver, and cholangitis. Shuganning injection selectively inhibited the proliferation of triple-negative breast cancer (TNBC) cells, and in in vivo studies confirmed that SGNI significantly suppresses the growth of TNBC xenografts and induces ferroptosis in nude mice [99].

Another so-called “heirloom recipe”, Formula X, contained *Coptis chinensis* Franch., *S. baicalensis* Georgi, *Vincetoxicum atratum*, *Curcuma aromatica* Salisb., and *Atractylodes macrocephala* Koidz. The authors revealed that Formula X could inhibit the growth of various cancer cells while reducing the expression of key proteins. A new formulation, BBS, containing berberine, baicalin, and saponin, effectively suppressed tumor growth in a mouse model [100].

Last but not least, Sanhuang Xiexin Decoction (SXD) was prepared by mixing *Rheum palmatum* L., *Scutellariae Radix*, and *Coptis chinensis* Franch in a 2:1:1 ratio. The bioactive compounds found in SXD were rhein, coptisine, berberine hydrochloride, and baicalin. The authors found that SXD demonstrated significant antitumor effects in 4T1 breast cancer mice through modulation of lipid profiles and cytokine levels, and low toxicity levels in mice were observed [101].

Plant extracts containing baicalein and baicalin exhibit significant anticancer potential in breast cancer models. According to the above-cited papers, these extracts inhibit cancer cell proliferation, induce apoptosis, and suppress tumor growth through diverse mechanisms. The variability in baicalein and baicalin content across extracts highlights the importance of standardized formulations for therapeutic applications. Despite the promising anticancer effects of plant extracts containing baicalein and baicalin, challenges in standardization remain a critical barrier to therapeutic translation. Variability in compound levels across species (e.g., *S. angulosa* vs. *S. baicalensis*), growth conditions, extraction methods (e.g., cellulase pretreatment in Yu et al. [97]), and formulation processes (e.g., SXD vs. HQT decoctions) complicates reproducibility. For instance, the baicalin-deprived fraction in Wang et al. [96] showed enhanced activity, highlighting how minor compositional differences significantly alter efficacy. Batch-to-batch inconsistencies in traditional formulations like SGNI [99] further underscore the need for rigorous quality control, phytochemical profiling, and harmonized protocols to ensure clinical reliability.

### 3.6. Derivatives of Baicalein in Anticancer Studies Against Breast Cancer

Baicalein demonstrates more substantial anticancer effects than baicalin because baicalein exhibits higher bioavailability compared to baicalin due to its structural characteristics, as it does not require hydrolysis for cellular uptake and its ability to interact with intracellular targets directly [103]. Furthermore, the clinical utility of baicalein is limited by its poor pharmacokinetic properties. The chemical modification of baicalein to create derivatives with improved activity and bioavailability presents a promising strategy to address these limitations. Over the last fifteen years, five papers have been published in which scientists attempted to obtain new baicalein derivatives and tested these compounds’ activity against breast cancer lines [104,105,106,107,108]. Table 5 provides a detailed overview of various baicalein derivatives and their effects on breast cancer cell lines.

**Figure 5:**
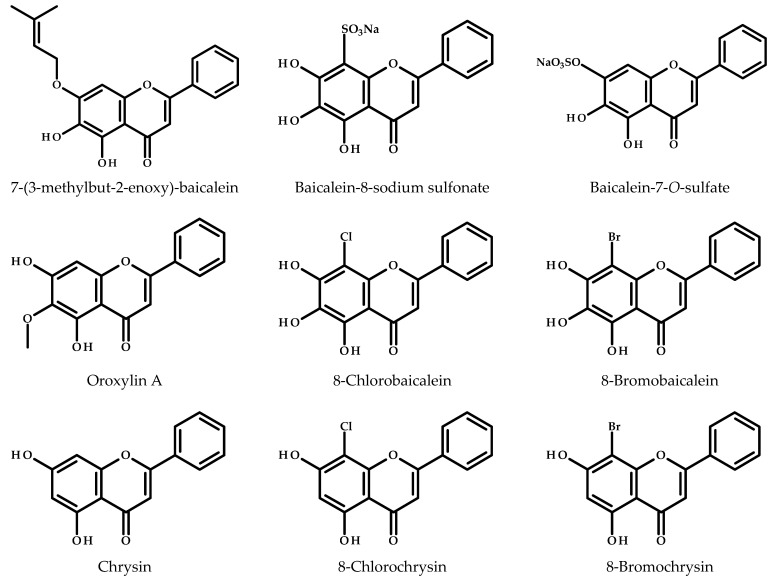

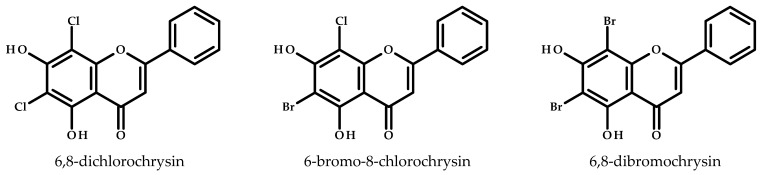
Chemical structures of baicalein derivatives.

One of them, 5,6-dihydroxy-7-(3-methylbut-2-enyloxy)- 2-phenyl-*4H*-chromen-4-one (prenylated baicalein, Figure 5), was synthesized by microwave-assisted organic synthesis from baicalein, prenyl bromide, and anhydrous potassium carbonate [104]. The obtained compound exhibited a dose-dependent inhibitory effect on MCF-7 and MDA-MB-231 cells. The observed activity was more potent in the case of MCF-7 cells and was mediated through interactions with estrogen receptors. Additionally, prenylated baicalein showed potential for synergistic effects with 4-hydroxytamoxifen and fulvestrant, suggesting its utility in combination therapies for estrogen receptor-positive breast cancer [104].

Two other sulfated derivatives, namely baicalein-7-*O*-sulfate and baicalein-8-sodium sulfonate, were investigated by Wang et al. [105] to evaluate their effects on the growth of MCF-7 cells and normal epithelial H184B5F5/M10 cells. The compounds showed cytotoxic effects only on MCF-7 cells, and baicalein-8-sodium sulfonate demonstrated the highest cytotoxic effect. Moreover, the obtained analogs induced apoptosis, increased intracellular ROS levels, and led to cell cycle arrest primarily at the G0/G1 phase.

Oroxylin A, a methylated metabolite of baicalein in the human body, was studied by An et al. [106]. Oroxylin A inhibited CYP1B1-mediated 4-hydroxylation of 17β-estradiol, a key process in estrogen metabolism, and was found to be more potent than baicalein [106].

Marzec et al. [107] designed a range of halogenated derivatives based on the natural flavonoids baicalein and chrysin. In the case of the former, it was found that halogenation increased the antiproliferative effects on different cell lines, such as LoVo, A549, MCF-7, and MCF-10A. Unfortunately, this indicates a lack of selectivity because they were also active against non-tumorigenic breast cells (MCF-10A). 8-Bromobaicalein was also studied by Yasuda et al. [108]. Its cytotoxic activity was evaluated, and the activity after its complexation with 2,6-di-*O*-methyl-β-cyclodextrin. The authors revealed that the inclusion complex of 8-bromobaicalein and cyclodextrin demonstrated significantly enhanced anticancer activity against MCF-7 human breast cancer cells compared to baicalein analog alone, with the IC_50_ values of 5.91 ± 0.35 and 15.53 ± 1.17 μM, respectively.

Chemical modifications, such as prenylation, sulfonation, halogenation, and complexation with cyclodextrins, have enhanced baicalein’s anticancer activity. These derivatives exhibit diverse mechanisms of action, including induction of apoptosis, cell cycle arrest, and modulation of oxidative stress. However, further research is needed to optimize their selectivity and minimize toxicity to non-cancerous cells.

Table 6 provides a detailed comparison between baicalin and baicalein, focusing on their molecular properties, pharmacokinetics [109], biological activities, and mechanisms of action. Baicalin, which has a molecular weight of 446.36 g/mol, features a sugar moiety that enhances its polarity. This is evidenced by its higher topological polar surface area (TPSA: 187.12 Å^2^) and hydrogen-bonding capacity (11 acceptors, 6 donors) compared to its aglycone counterpart, baicalein. The latter also exhibits greater lipophilicity (LogP 2.24 vs. 0.25). Baicalin’s high polarity results in low water solubility (−3.41 LogS) and reduced gastrointestinal (GI) absorption, further affected by its status as a P-glycoprotein (P-gp) substrate. Conversely, baicalein’s lipophilic nature enhances GI absorption despite its lower solubility. Although neither compound crosses the blood-brain barrier (BBB), baicalein has better skin penetration (Log Kp −5.70 compared to −8.23 for baicalin). Importantly, baicalein inhibits cytochrome P450 enzymes (CYP1A2, CYP2D6, CYP3A4), which raises concerns for potential drug interactions, whereas baicalin does not inhibit CYP enzymes.

### 3.7. Synergistic Effects of Baicalin/Baicalein in Combination Therapies for Breast Cancer

Combination therapies have emerged as a promising strategy for enhancing the efficacy of anticancer treatments while minimizing side effects. By utilizing multiple agents with complementary mechanisms of action, these therapies offer a robust approach to overcoming the limitations associated with traditional single-agent treatments, thereby improving cancer patients’ outcomes. The outcomes of combining baicalin and baicalein with other therapeutic agents focused on their synergistic and antagonistic effects in breast cancer treatment are presented in Table 7 [110,111,112,113,114,115,116,117,118,119,120,121,122,123].

Baicalin, together with *Salvia miltiorrhiza* extract, was the first combination documented in scientific literature and utilized in breast cancer cell lines [110]. Their combination resulted in synergistic effects, significantly enhancing the inhibition of breast cancer cell lines (MCF-7 and T-47D) as well as other cancer types (FaDu and CAL-27). Similarly, Xi et al. [114] found that the combination of baicalein and low-frequency ultrasound (5 min at 840 kHz, 0.75 W/cm^2^) demonstrated synergistic effects in reducing the invasive activity of triple-negative breast cancer cells (MDA-MB-231). The invasive capacity of cells in the low-frequency group, baicalein group, and combination group was reduced by 21.6%, 31.9%, and 51.6%, respectively. This was achieved by downregulating the expression of MMP-2, MMP-9, and urokinase-type plasminogen activator (u-PA), key mediators of cancer cell invasion and metastasis [114].

Baicalin and baicalein have been shown to mitigate the toxic side effects of chemotherapy agents. Chang et al. [111] found that baicalein protected against doxorubicin-induced cardiotoxicity by reducing ROS generation and preserving mitochondrial function in chick cardiomyocytes without interfering with doxorubicin’s anticancer activity in MCF-7 cells. In vivo studies have also shown promising results. Zeng et al. [116] demonstrated that baicalin enhanced the chemosensitivity of MDA-MB-231 cells to docetaxel, inhibiting tumor growth and pulmonary metastasis in female BALB/c mice. Additionally, Shehatta et al. [118] reported that the combination of baicalin and 5-fluorouracil significantly inhibited tumor growth and promoted apoptosis in Swiss albino mice. Similarly, Ibrahim et al. [120] reported that baicalein partially mitigated capecitabine-induced cardiotoxicity, oxidative stress, and inflammation in adult female albino rats.

Not all combinations of baicalin or baicalein with other agents yield synergistic effects. Zhan et al. [112] observed that the combination of baicalein and paclitaxel resulted in antagonistic effects, reducing the growth-inhibitory effect of paclitaxel in MCF-7 cells.

The mechanisms underlying the synergistic effects of baicalin and baicalein in combination therapies are diverse. An et al. [113] demonstrated that the combination of baicalein with U0126 enhanced cell proliferation inhibition, apoptosis induction, and migration suppression in MCF-7 cells, likely through modulation of the MAPK/ERK signaling pathway. Lin et al. [118] reported that baicalin enhanced doxorubicin’s anticancer effects by increasing oxidative stress, calcium levels, and mitochondrial dysfunction in breast cancer cells. Furthermore, Hua et al. [121] found that baicalein enhanced the sensitivity of MDA-MB-231 cells to doxorubicin by activating autophagy and mitophagy.

Recent studies by Sisin et al. [115,119] have explored novel combinations involving bismuth oxide nanoparticles, cisplatin, and a baicalein-rich fraction from *O. indicum*. The combination treatment was highly effective in breast cancer cells, demonstrating the most potent enhancement of ROS generation and radiosensitization across all types of radiation tested. However, it also showed significant radiosensitization effects in normal cells when a baicalein-rich fraction was added, which could impact safety profiles [115,119]. Awajan et al. [123] reported that the combination of baicalin, epigallocatechin gallate (EGCG), and vincristine had synergistic effects in vincristine-resistant cancer cells, significantly reducing tumor size and achieving high cure rates in female BALB/c mice.

### 3.8. Nanotechnology-Based Delivery Systems for Baicalin/Baicalein in Breast Cancer Research

Nanotechnology presents a promising solution to the challenges associated with traditional drug delivery systems by enabling targeted delivery. Nanoparticles can enhance therapeutic agents’ solubility, stability, and bioavailability while minimizing off-target effects [124]. This approach has been explored for various compounds, including baicalein and baicalin [125,126,127,128,129,130,131,132,133,134,135,136]. Scientists have used, e.g., iron oxide and gold nanoparticles, nanoemulsions, nanostructured lipid carriers, nanocapsules, and Metal-Organic Frameworks (MOFs) (Table 8).

Kavithaa et al. [125,129] demonstrated that baicalein-loaded iron oxide nanoparticles induced significant cytotoxicity in MDA-MB-231 cells (IC_50_ = 22 µg/mL) by disrupting the cell cycle and promoting apoptosis. The nanoparticles upregulated pro-apoptotic proteins (Bax, cytochrome-c, caspase-3, PARP, and p53) while downregulating the anti-apoptotic protein Bcl-2. Similar findings were reported by Srivastava et al. [133], who developed a baicalein and cinnamon essential oil nanoemulsion that exhibited 19-fold and 23-fold higher cytotoxicity compared to free baicalein after 12 and 24 h, respectively. This enhanced activity was attributed to improved drug permeability and cellular uptake via endocytosis.

Baicalin was also applied in nano-delivery systems. Lee et al. [126] developed gold nanoparticles (AuNPs) conjugated with thiolated beta-cyclodextrin for baicalin delivery, significantly enhancing its apoptotic effects in MCF-7 cells. Similarly, El-Gogary et al. [130] reported that baicalin-loaded polylactide-glycolide (PLGA) nanocapsules demonstrated superior anticancer activity, sustained drug release, and enhanced cellular uptake in MCF-7 and MDA-MB-231 cells.

Several studies have explored the synergistic effects of baicalein combined with other therapeutic agents. Meng et al. [127] developed a baicalein and paclitaxel nanoemulsion that showed enhanced anticancer efficacy in MCF-7 and MCF-7/Tax cells. The nanoemulsion achieved a tumor inhibition rate of 77.0% in an MCF-7/Tax xenograft model, with minimal weight loss in treated mice. Liu et al. [128] reported similar findings for a hyaluronic acid-decorated nanostructured lipid carrier (NLC) co-delivering baicalein and doxorubicin, which exhibited a tumor inhibition rate of 88% in Kunming mice.

Mi et al. [131] designed a methoxy poly(ethylene glycol)-folic acid-decorated zeolitic imidazolate framework (ZIF-8) for baicalin delivery, which demonstrated excellent tumor-targeting capability and pH-responsive drug release. Similarly, Meng et al. [132] and Liu et al. [135] developed folate-modified albumin nanoparticles for baicalin delivery, which showed sustained drug release, enhanced cellular uptake, and potent anticancer activity in vitro and in vivo.

Finally, green synthesis of nanoparticles using plant extracts has also been explored. Gharari et al. [136] synthesized silver nanoparticles using *S. multicaulis* leaf extract, demonstrating significant antioxidant, anticancer, and apoptotic activities in MDA-MB-231 cells.

## 4. Conclusions

**Figure 6:**
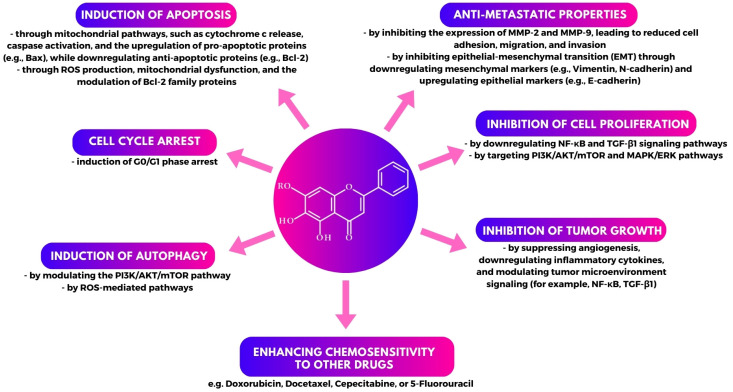
An overview of the key mechanisms behind the anticancer effects of baicalin and baicalein. R represents either a hydrogen atom (H) for baicalein or a glucuronide moiety for baicalin.

This systematic review thoroughly evaluates the roles of baicalin and baicalein in breast cancer, emphasizing their therapeutic potential through various mechanisms. Both flavonoids demonstrate significant antiproliferative, pro-apoptotic, and anti-metastatic effects across different breast cancer models. Baicalin and baicalein regulate key pathways, including apoptosis (via caspase activation and Bcl-2 family regulation), autophagy, oxidative stress (ROS modulation), and inflammation (NF-κB suppression) (Figure 6).

Plant extracts from *S. baicalensis* and other species enhance anticancer effects through synergistic interactions, while chemical derivatives (e.g., sulfonated, halogenated, and cyclodextrin-complexed forms) increase efficacy and selectivity. Combination therapies with conventional agents like doxorubicin or docetaxel produce synergistic outcomes, reducing chemotherapy resistance and toxicity. Nanotechnology-based delivery systems, such as lipid carriers and metal-organic frameworks, tackle bioavailability challenges and improve tumor-targeted delivery.

Despite encouraging preclinical outcomes, clinical translation remains limited. Variability in responses across breast cancer subtypes and potential off-target effects in non-cancerous cells highlight the need for further optimization. Future research should focus on large-scale, randomized clinical trials to validate efficacy across breast cancer subtypes, especially in resistant or metastatic situations. Standardized formulations with defined ratios of baicalein/baicalin (e.g., enhanced through nanotechnology [125,126,127,128,129,130,131,132,133,134,135,136]) and advanced delivery systems (e.g., tumor-targeted nanoparticles, cyclodextrin complexes [108]) could improve bioavailability and minimize off-target effects. Additionally, investigating synergistic combinations with immunotherapy or targeted therapies, along with mechanistic studies on subtype-specific responses (e.g., ER+ vs. TNBC), may reveal personalized therapeutic strategies. Addressing these gaps will connect preclinical promise to clinical utility. Collectively, baicalin and baicalein emerge as promising candidates for adjuvant or standalone therapies, warranting continued exploration in the evolving landscape of breast cancer treatment.

## Figures and Tables

**Table 1:** The five scientific publications most frequently cited in the Scopus database that reference baicalin and baicalein.

Rank	Authors	Title	Year	Journal	Total Citation	Reference
1.	Cushnie, T.P.T.; Lamb, A.J.	Antimicrobial Activity of Flavonoids	2005	*International Journal of Antimicrobial Agents*	3287	[31]
2.	Del Rio, D.; Rodriguez-Mateos, A.; Spencer, J.P.E.; Tognolini, M.; Borges, G.; Crozier, A.	Dietary (Poly)phenolics in Human Health: Structures, Bioavailability, and Evidence of Protective Effects Against Chronic Diseases	2013	*Antioxidants and Redox Signaling*	2067	[32]
3.	Wu, C.; Liu, Y.; Yang, Y.; Zhang, P.; Zhong, W.; Wang, Y.; Wang, Q.; Xu, Y.; Li, M.; Li, X.; et al.	Analysis of Therapeutic Targets for SARS-CoV-2 and Discovery of Potential Drugs by Computational Methods.	2020	*Acta Pharmaceutica Sinica B*	1712	[33]
4.	Formica, J.V.; Regelson, W.	Review of the Biology of Quercetin and Related Bioflavonoids.	1995	*Food and Chemical Toxicology*	1617	[34]
5.	Zhang, Q.-W.; Lin, L.-G.; Ye, W.-C.	Techniques for Extraction and Isolation of Natural Products: A Comprehensive Review	2018	*Chinese Medicine*	1331	[35]

**Table 2:** Overview of studies on the anticancer potential of baicalin.

Cell Lines	Animal Models	Tested Concentrations	The Outcome of the Study	Reference
PC-3, DU145, LNCaP, MCF-7, HL-60, and NB4	-	0.1–20 µM	The MCF-7 cell line was insensitive to growth inhibition by baicalin	[36]
MCF-7	-	50–400 µM	Inhibition of MCF-7 cell proliferation in a dose- and time-dependent manner (IC_50_ = 206 µM after 24 h) At 200 µM baicalin induced potent cytotoxicity and morphological changes indicative of cell death Cells arrested in G0/G1 phase and chromatin condensation confirmed apoptosis Upregulation of protein p53 and the bax gene	[37]
MCF-10A and MDA-MB-231	-	2–5 µM	Inhibition of NF-κB activation and TGF-β1-induced EMT in both cell lines Suppression of TGF-β1-induced migration and colony formation in MDA-MB-231 breast cancer cells	[38]
MCF-7	-	10–160 µM	Baicalin at 160 µM significantly reduced the survival of MCF-7 cells, and concentrations of 10–80 µM had no significant effect Inhibition of MCF-7 cell migration by upregulating miR-200b and E-cadherin	[39]
MDA-MB-231 and 4T1	-	10–100 µM	Baicalin (up to 100 µM) did not affect the viability of MDA-MB-231 and 4T1 cells Baicalin suppressed breast cancer metastasis by inhibiting migration, invasion, and EMT through the β-catenin pathway	[40]
MCF-7, MDA-MB-231, and MCF-10	Female BALB/c nude mice (5 weeks old)	20–30 µM	Inhibition of breast cancer cell proliferation, invasion, and migration by inducing G1/S arrest and suppressing the NF-κB pathway Reduction of tumor growth in vivo and modulated inflammatory responses	[41]
MDA-MB-231	-	10–40 µg/mL	Inhibition of MDA-MB-231 cell growth, proliferation, migration, and invasion, with effects increasing at higher concentrations (40 μg/mL was the most effective) Reducing Vimentin, β-catenin, c-Myc, and MMP-7 mRNA expression while increasing E-cadherin expression	[42]
MCF-10A, MCF-7, and MDA-MB-231	-	25–200 µM	Inhibition of viability, migration, and invasion of breast cancer cells, but no cytotoxicity to normal MCF-10A cells Inhibition of breast cancer progression by upregulating miR-338-3p	[43]
SK-BR-2 and MCF-7	Female BALB/c-null and normal mice (6 weeks old)	100 mg/kg	Baicalin bonded directly to TGF-β1 and downregulated its expression, along with p-Smad3 and vimentin, while upregulating E-cadherin Baicalin suppressed tumor growth and metastasis, with upregulated E-cadherin and downregulated TGF-β1, vimentin, and p-Smad3 in tumors	[44]
MDA-MB-231 and MCF-7	-	0.25–100 nM	Induction of apoptosis in breast cancer cells (MDA-MB-231, MCF-7) by increasing cytochrome c release, DNA fragmentation, and caspase-3, -8, and -9 activity Inhibition of the mTOR pathway, reducing phosphorylation of mTOR and p70 S6 kinase—Baicalin had minimal effects on osteoblasts and bone cells	[45]
MCF-7	-	50–200 µM	Inhibition of MCF-7 cell proliferation in a time- and concentration-dependent manner (0–200 µM, 24–72 h) At 150 µM baicalin, miRNA microarray analysis identified 92 upregulated and 35 downregulated miRNAs	[46]
Eph4, MDA-MB-231, ZR-75-1, and 293T	C57BL/6 and Actin-DsRed mice (3 weeks old)	50–200 mg/kg	Baicalin suppressed luminal breast cancer (ZR-75-1) growth by downregulating ESR1 but promoted triple-negative breast cancer (MDA-MB-231) growth by upregulating PROCR Baicalin exhibited steroid hormone-like activity, regulating genes involved in Hippo signaling and cell cycle while suppressing breast cancer-related genes	[47]
MADB-106	Sprague-Dawley (SD) rats	30 mg/kg	Reversing bone loss in bone cancer pain rats Inhibition of osteoclast activation and reduction of inflammation Downregulating cancer-related genes and modulating pathways like NF-κB and TNF signaling.	[48]
MDA-MB-231	-	12.5–50 µM	Reduction of cell viability of MDA-MB-231 cells after 48–72 h of treatment Inhibition of migration and invasion of breast cancer cells Downregulation of the TGF-β/ZEB1 pathway, reducing TGF-β1, ZEB1, and N-cadherin (mesenchymal markers) while upregulating E-cadherin (epithelial marker) at both mRNA and protein levels Downregulation of lncRNA-MALAT1 and upregulated miR-200c	[49]
MCF-7	-	0–200 µM	Anti-proliferative and cytotoxic activity against breast cancer cells (IC_50_ = 34.8 μM) Baicalin triggered apoptosis and heightened ROS production in MCF7 cells	[50]
MCF-7, MDA-MB-231, MCF-7 ADRB2^OE	Female BALB/c and C57BL/6J mice (6 weeks old)	25–100 µM	Baicalin suppressed epinephrine-induced migration and invasion of breast cancer cells in wound healing, transwell, and 3D spheroid assays Protection against chronic stress-promoted breast cancer metastasis by binding to β2-AR and blocking its activation on tumors	[51]

**Table 3:** Summarized data on baicalein application in breast cancer models.

Cell Lines	Animal Models	Tested Concentrations	The Outcome of the Study	Reference
MDA-MB-435	Female Sprague-Dawley rats	0–250 µg/mL	Inhibition of MDA-MB-435 breast cancer cell proliferation in vitro, with an IC_50_ of 5.9 µg/mL	[52]
MCF-7	-	0–2 mg/mL	Baicalein inhibits the proliferation of MCF-7 breast cancer cells through a non-estrogenic mechanism (IC₅₀ = 5.3 µg/mL)	[53]
Caco-2, HT-29, LLC-PK1, and MCF-7	-	1–1000 µM	MCF-7 breast cancer cells were less sensitive to baicalein, requiring 6.6-fold higher concentrations to achieve 50% growth inhibition compared to intestinal cells	[54]
MCF-7 and MDA-MB-231	-	1.25–20 µM	Inhibition of the proliferation of both MDA-MB-231 and MCF-7 breast cancer cells Induction of apoptosis through mitochondrial pathways, caspase activation, and modulation of Bcl-2 family proteins	[55]
MCF-7	-	10–100 µM	Induction of apoptosis in MCF-7 cells at 10 µM Baicalein antagonized estradiol-induced ER transactivation, showing consistent antiestrogenic effects	[56]
MCF-7	-	5 µM	Inhibition of the proliferative effects of THF-diols and E2 on MCF-7 breast cancer cells by targeting LOX-12	[57]
MCF-7	-	25–50 µM	Baicalein, but not its glycoside inhibited E2/IGF-I-induced proliferation, c-Jun expression, and ROS production	[58]
4T1	-	1–100 µM	Demonstration of anti-proliferative, anti-metastatic, and antioxidant properties in 4T1 tumor spheroids	[59]
MDA-MB-231	-	25–100 µM	Induction of apoptosis in MDA-MB-231 breast cancer cells through ROS production, mitochondrial dysfunction, Ca^2+^ signaling, and regulation of apoptotic proteins	[60]
MDA-MB-468 and MCF-10A	-	10 µM	Antiproliferative effects in breast cancer cells (MDA-MB-468), Baicalein was not metabolized by CYP1 enzymes	[61]
MCF-7 and CCRFCEM leukemia cells	-	1–1000 µM	Baicalein (and baicalin) induced G1-phase arrest in MCF-7 and CCRFCEM cells—Moderate anticancer activity (IC_50_ = 69.6 μM for baicalein and 78.8 μM for baicalin against MCF-7 cells) Baicalein-induced DNA damage in MCF-7 cells (Baicalin had no effect)	[62]
MCF-7 and MDA-MB-231	-	25–400 µM	The combination of baicalin and baicalein synergistically inhibited breast cancer cell growth, induced apoptosis, and caused cell cycle arrest by activating the ERK/p38 MAPK pathway	[63]
MDA-MB-231	-	2–50 µM	Baicalein demonstrates potent anti-metastatic effects by inhibiting adhesion, motility, and invasion of MDA-MB-231 breast cancer cells Suppression of the activity and expression of MMP-2 and MMP-9, likely through the downregulation of the MAPK signaling pathway	[64]
AGS, MDA468, BT549, SKBR3, C3L5, and MDA468-C23	Female SCID-Bg mice (Charles River, 6 weeks old)	0.5–10 µM	Baicalein enhanced IRF-1 activity, upregulated tumor-suppressive pathways, and inhibited cancer cell growth both in vitro and in vivo	[65]
MCF-7 and breast cancer stem cells (CSCs)	-	0–120 µM	Baicalein exhibits limited effectiveness against breast CSCs compared to ginsenoside F2	[66]
MCF-7	-	100 µM	Inhibition of ALOX12/15 and reduction of 12(S)-HETE production	[67]
PC-3, MDA-MB-231 and DU145	-	2.5–20 µg/mL	Induction of autophagy, dysfunction of mitochondria, and cell cycle arrest	[68]
MDA-MB-468, SKBR3, Hs578T, and BT549	SCID-Beige mice	5–80 µM	Baicalein-induced DDIT4 (DNA Damage-Inducible Transcript 4) expression in multiple breast cancer cell lines Baicalein treatment significantly suppressed tumor growth without toxicity in an orthotopic mouse model of triple-negative breast cancer	[69]
ZR-75-1	-	10–40 µM	Baicalein increased intracellular Ca^2+^ concentration through Ca^2+^ release from the ER and Ca^2+^ entry via SOCCs, leading to ROS production, caspase activation, and apoptosis in ZR-75-1 cells	[70]
MDA-MB-231	-	5–80 µM	Inhibition of proliferation and migration of MDA-MB-231 cells Downregulation of SATB1 protein expression	[71]
MCF-7 and SK-BR-3	-	5–50 µM	Inhibition of E2-induced migration, adhesion, invasion, and GPR30 signaling in breast cancer cells	[72]
MCF-10A, MCF7, SKBR3, and MDA-MB-231	Female Balb/c nude mice (4–8 weeks old)	10–40 µM	Suppression of proliferation, migration, and invasion of cancer cells Downregulation of SATB1 and the Wnt/β-catenin pathway Reversing EMT and reducing metastasis in vivo	[73]
MCF-7	-	10 µM	Inhibition of E2-induced invasion and MMP-9 activity in MCF-7 breast cancer cells by targeting GPR30 signaling	[74]
A549, MCF-7, and U87	-	3.125–100 µM	Moderate anti-proliferative activity against the MCF-7 breast cancer cell line (EC_50_ = 26.1 µM)	[75]
MCF-10A and MCF-12A	Female non-obese diabetic/severe combined immunodeficiency (NOD/SCID) mice (6–9 weeks old)	2–8 µM	Baicalein effectively prevents estradiol (E2)-induced neoplastic transformation in mammary epithelial cells by inhibiting cell growth, migration, invasion, and tumorigenesis, and by blocking ERα and GPR30 signaling pathways	[76]
MDA-MB-231 and MCF-7	Seventy-six pairs of breast cancer and adjacent normal tissues from patients after surgery	50–400 µM	Upregulation of PAX8-AS1-N, resulting in the inhibition of tumor growth and progression by modulating miR-17-5p and its downstream targets	[77]
MCF-7 and MDA-MB-231	Female BALB/c nude mice (3–6 weeks old)	10–40 µM	Inhibition of proliferation and induction of apoptosis and autophagy Suppression of the PI3K/AKT/mTOR signaling pathway	[78]
MDA-MB-231, MCF-10A, and HeLa	-	2–50 µM	Inhibition of breast cancer cell motility by disrupting paracrine interactions mediated by laminin-332	[79]
MDA-MB-231 and MCF-7	-	20 µM	Baicalein inhibited oleic acid-induced migration and invasion in breast cancer cells Blocking of AKT2 and FAK activation Reduction of 12(S)-HETE secretion through 12-LOX inhibition Altering focal contact formation	[80]
MDA-MB-231 and MDA-MB-231/IR	-	20–100 µM	Reversion of IFIT2 expression, suppression of stem cell-like properties, and induction of apoptosis	[81]
MCF-10A	Female MMTV-PyMT transgenic mice (about 3 weeks old)	2.5–10 µM	Inhibition of fibronectin-induced migration, invasion, and epithelial-mesenchymal transition in breast epithelial cells by suppressing calpain-2 activity and modulating Ca^2+^ levels and ERK signaling In vivo, baicalein delays tumor progression, reduces metastasis, and prolongs survival in a breast cancer mouse model	[82]
MCF-7 and MCF-10A	-	12.5–200 µM	Inhibition of MCF-7 breast cancer cell proliferation and induction of apoptosis through a copper-dependent mechanism involving ROS generation and mitochondrial dysfunction	[26]
MCF-7	-	10–75 µM	Exhibition of weak activity in the CCID assay (IC_50_ = 130.2 µM)	[83]
4T1 and MDA-MB-231	-	10–100 µM	Baicalein suppressed STAT3 activity by inhibiting its phosphorylation and reduces IL-6 production, leading to anti-proliferative, cytotoxic, and anti-metastatic effects	[84]
MCF-7	-	20 µM	Antiproliferative effect on MCF-7 cells Inhibition of TNF-α and IL-10	[85]
B16-F10, 4T1, THP-1	-	20–80 µM	Baicalein exerted its anti-tumor effects by promoting M1 macrophage polarization through the NF-κB/TNF-α signaling pathway	[86]
MCF-7	-	20–84 µM	Exhibition of the cytotoxicity in MCF-7 cells (CC₅₀ = 56.46 µM)—Baicalein inactivated free virus particles and modestly reduced MV infection when added concurrently or immediately post-infection	[87]
MDA-MB-231, BT549, MDA-MB-468, MCF-7, ZR-75-1, T47D, and MCF-10A	BALB/c nude mice (6–8 weeks old)	10–100 µM	Anti-tumor effects by targeting the KDM4E/BICD1/PAR1 signaling pathway	[88]
MDA-MB- 231, BT549, 4T1, and 3T3-L1	Female BALB/C mice (4 weeks old)	10–40 µM	Baicalein significantly slowed tumor growth and downregulated PD-L1, LEP, SREBP1, and p-STAT3 in tumors	[89]
MCF-7	-	25–400 µM	Inhibition of MCF-7 cells viability, induction of apoptosis, and suppression of the migration and invasion by modulating the Wnt3α/β-catenin signaling pathway Upregulation of Nischarin	[90]
MDA-MB-231, MCF-7, and HeLa	-	3.23–22.2 µM	Induction of LDH release and increased caspase-3-activity, indicating cell membrane damage and apoptosis	[91]

**Table 4:** Summary of the studies investigating plant extracts containing baicalein and baicalin for anticancer activity in breast cancer models.

Cell Lines	Animal Models	Plant/Materials	Baicalein Content	Baicalin Content	The Outcome of the Study	Reference
U87-MG, U251, MDA-MB-231, HMEC, and PC3	-	thirteen *Scutellaria* species	0.21–2.34 μg/mg extract	0.92–25.34 μg/mg extract	*Scutellaria* species inhibited the proliferation of MDA-MB-231 cells and induced apoptosis	[19]
MDA-MB-435S	-	*Phyllanthus amarus* (whole plant)	present	not specified	The application of the aqueous extract mitigated Cr(VI)-induced oxidative toxicity in MDA-MB-435S in a dosage-dependent manner	[20]
MCF-7	-	the roots of *S. baicalensis*	40.54% in the baicalin-deprived fraction, 0.00–14.24% in other fractions	0.51–19.58%	The presence of baicalin in the isolated fractions reduced their antiproliferative effects Baicalin-deprived fraction primarily contained baicalein, and wogonin demonstrated the strongest anticancer activity, primarily through cell cycle arrest and apoptosis induction	[96]
HCT-116 and MCF-7	-	*S. baicalensis* root powder	8.5–68.6%	1.0–43.3%	Cellulase treatment significantly enhanced the antiproliferative potential of *S. baicalensis* extracts against cancer cells and promoted apoptosis through S-phase cell cycle arrest	[97]
MCF-7, HeLa, HT-29, and MRC-5	-	black (*Tuber aestivum* Vittad.) and white (*T. magnatum* Pico) truffles	2.01–15.73 µg/g dw in *T. aestivum*	-	LC-MS/MS analysis identified 14 phenolic compounds, with *T. aestivum* rich in *p*-hydroxybenzoic acid, baicalein, and kaempferol, and *T. magnatum* in epicatechin and catechin *T. aestivum* and *T. magnatum* extracts showed significant growth inhibition in MCF-7 and HeLa cells	[21]
MCF-7	-	*S. baicalensis* Georgi extract	not specified	present	The extract exhibited potent anti-cancer effects on MCF-7 cells through mechanisms involving apoptosis induction via mitochondrial pathways, caspase activation, and ROS generation	[98]
4T1	-	leaves of *Vernonia amygdalina* Delile	present	not present	Ethyl acetate fraction of *V. amygdalina* Delile demonstrated anticancer potential by inducing cell cycle arrest, apoptosis/necrosis, and inhibiting PI3K/mTOR pathways	[22]
SK-BR-3, MDA-MB-231, MDA-MB-468, BT-549, MCF10A, 786-O, A2780, HCT116, HepG2, A549, Lo2, and WPMY-1, U-87 MG, and HT1080	Female nude mice (4–6 weeks old)	Shuganning injection	not specified	not specified	It inhibited TNBC cell proliferation more effectively than non-TNBC cells It induces non-apoptotic, lipid peroxidation-dependent cell death (ferroptosis) In vivo experiments with nude mice showed that SGNI significantly inhibited tumor growth without adverse effects	[99]
MCF-7, Huh7, T47D, HCC 1954, AGS, LoVo, Mia-Paca2, U2OS, and MDA-MB-231	Female C57BL/6 mice (6 weeks of age)	“Heirloom recipe” (Formula X)	not present	5.07 mg/g	Formula X suppressed the growth of four breast cancer cell lines (T47D, MCF7, MDA-MB231, HCC1954) in a dose-dependent manner Reduction of the expression of the oncogenic proteins Berberine, baicalin, and saponin mixture reduced tumor growth in mouse models	[100]
MCF-7, T47D, Vero, and EMT6/P	BALB/c female mice (4–6 weeks old)	*Xanthium spinosum*	present in methanol fraction	present in hexane fraction	In vivo studies showed significant tumor size reduction in mice treated with *X. spinosum* extracts	[23]
4T1	Female BALB/c mice (4–6 weeks old)	Sanhuang Xiexin Decoction (SXD)	not present	46.04 mg/g	SXD demonstrated significant antitumor effects against 4T1 breast cancer in mice through modulation of lipid profiles, cytokine levels, angiogenesis inhibition, and low toxicity	[101]
MCF-7 and MDA-MB-231	-	Huangqin Tang (HQT)	present	not specified	HQT inhibited proliferation, induced apoptosis, induced G2/M phase arrest, and reduced HIF-1α protein levels in breast cancer cells	[102]
MCF-7, MDA-MB-231, SKBR-3, and L929	-	aerial parts of *Centaurea mersinensis*	11.12 µg/g	648.28 µg/g	The methanol extract showed moderate cytotoxicity against breast cancer cell lines (IC_50_ = 22.17–46.20 µg/mL)	[24]

**Table 5:** Derivatives of baicalein and their anticancer effects in breast cancer research.

Derivative(s)	Cell Lines	Animal Models	The Outcome of the Study	Reference
Prenylated baicalein	MCF-7 and MDA-MB-231	-	The prenylated baicalein derivative demonstrated antiproliferative activity in MCF-7 cells, likely mediated through estrogen receptor interactions, and showed potential for synergistic effects with 4-hydroxytamoxifen and fulvestrant	[104]
Baicalein-8-sodium sulfonate and Baicalein-7-*O*-sulfate	MCF-7 and H184B5F5/M10	-	Baicalein-8-sodium sulfonate exhibited a higher anticancer effect than its precursor, induced apoptosis, cell cycle arrest, and ROS-mediated oxidative stress in MCF-7 cells	[105]
Oroxylin A	MCF-7	-	Oroxylin A inhibited CYP1B1-mediated 4-hydroxylation of 17β-estradiol Oroxylin A was more potent than baicalein	[106]
8-Chlorobaicalein, 8-bromobaicalein, chrysin, 8-chlorochrysin, 8-bromochrysin, 6,8-dichlorochrysin, 6-bromo-8-chlorochrysin, and 6,8-dibromochrysin	A549, LoVo, MV4-11, MCF-7, and MCF-10A	-	Baicalein and its halogenated derivatives showed increased cytotoxicity towards breast cancer cells (MCF-7) but were also toxic to non-tumorigenic breast cells (MCF-10A)	[107]
8-Bromobaicalein	MCF-7	-	8-bromobaicalein and 2,6-di-O-methyl-β-cyclodextrin complexes demonstrated enhanced cytotoxic activity against MCF-7 breast cancer cells, with IC_50_ values significantly lower than free 8-bromobaicalein	[108]

**Table 6:** Comparative analysis of baicalin and baicalein in breast cancer models.

Property/Activity	Baicalin	Baicalein
Molecular Formula	C_21_H_18_O_11_	C_15_H_10_O_5_
Molecular Weight (g/mol)	446.36	270.24
H-Bond Acceptors	11	5
H-Bond Donors	6	3
Molar Refractivity	106.72	73.99
TPSA (Å^2^)	187.12	90.90
Consensus LogP_o/w_	0.25	2.24
Water Solubility LogS	−3.41	−4.03
GI Absorption	Low	High
BBB Permeant	No	No
P-gp Substrate	Yes	No
CYP Inhibition	None	CYP1A2, CYP2D6, CYP3A4
Skin Permeation (Log K_p_) (cm/s)	−8.23	−5.70
Breast Cancer Cell Lines Tested	4T1, MADB-106, MCF-7, MCF-7 ADRB2^OE, MDA-MB-231, SK-BR-2, ZR-75-1	4T1, BT549, Hs578T, MCF-7, MDA-MB-231, MDA-MB-231/IR, MDA-MB-435, MDA-MB-468, MDA-MB-468-C23, SK-BR-3, T47D, ZR-75-1
Bioavailability Issues	Poor water solubility Poor intestinal absorption	Better absorption than baicalin Rapid glucuronidation in liver/enterocytes
Derivatives	No derivatives were examined	Prenylated, sulfonated, and halogenated derivatives improved efficacy
Anti-Proliferative	Suppressed cell growth via ESR1 downregulation	Inhibition of cells via mitochondrial pathways and DDIT4 induction
Apoptotic	p53/Bax upregulation, caspase-3/-8/-9 activation, and ROS production	Activated mitochondrial apoptosis, caspase cascades, and Bcl-2 modulation, and increased Ca^2+^, ROS, and DNA damage
Anti-Metastatic	Inhibited EMT via β-catenin/TGF-β	Blocked MMP-2/9, SATB1/Wnt pathways
Pathway Targets	NF-κB, mTOR, miRNAs	STAT3, PI3K/AKT/mTOR, Wnt/β-catenin

**Table 7:** Combination therapies of baicalin/baicalein with other drugs in anticancer research against breast cancer.

Combination of:	Cell Lines	Animal Models	The Outcome of the Study	Reference
Baicalin and *Salvia miltiorrhiza* extract	MCF-7, T-47D, FaDu, and CAL-27	-	Combining baicalin with *S. miltiorrhiza* extract resulted in synergistic effects, significantly enhancing the inhibition of MCF-7, T-47D, and CAL-27 cells	[110]
Baicalin and Doxorubicin	MCF-7 and chick cardiomyocytes	-	Baicalein protected against doxorubicin-induced cardiotoxicity by reducing ROS generation, preserving mitochondrial function, and inhibiting JNK-mediated apoptosis Baicalein did not interfere with doxorubicin anticancer activity in MCF-7 cells	[111]
Baicalein and Paclitaxel	MCF-7	Female Kunming mice (4–6 weeks old)	When combined with paclitaxel, baicalein showed antagonistic effects, i.e., reduced the growth-inhibitory effect of paclitaxel	[112]
Baicalein and U0126	MCF-7	-	The combination of baicalein with U0126 enhanced inhibition of cell proliferation, induction of apoptosis and cell cycle arrest, and suppression of migration, likely through modulation of the MAPK/ERK signaling pathway	[113]
Baicalein and ultrasounds	MDA-MB-231	-	Synergistic effect of the combination of baicalein and ultrasound Both baicalein and low-frequency ultrasound effectively reduce the invasive activity of MDA-MB-231 cells by downregulating the expression of MMP-2, MMP-9, and u-PA at both the mRNA and protein levels	[114]
Bismuth Oxide Nanoparticles, Cisplatin, and Baicalein-Rich Fraction from *O. indicum*	MCF-7, MDA-MB-231, and NIH/3T3	-	The combination of bismuth oxide nanoparticles and cisplatin was the most effective in enhancing ROS generation and radiosensitization in breast cancer cells The addition of a baicalein-rich fraction had significant radiosensitization effects in normal cells	[115]
Baicalin and Docetaxel	MDA-MB-231, MDA-MB-453, and 4T1	Female BALB/c mice (6–8 weeks old)	Baicalin inhibited cell proliferation, induced mitochondria-mediated apoptosis, suppressed migration and invasion via the NF-κB pathway, and enhanced chemosensitivity to docetaxel Baicalin inhibited tumor growth and pulmonary metastasis	[116]
Baicalin and Doxorubicin	MDA-MB-231 and MCF-7	-	Baicalin enhanced doxorubicin anticancer effects by increasing oxidative stress, calcium levels, and mitochondrial dysfunction in breast cancer cells	[117]
Baicalin and 5-fluorouracil	-	Swiss albino mice	The combination of baicalin and 5-fluorouracil demonstrated the most effective antitumor activity, significantly inhibiting tumor growth, promoting apoptosis, and reducing tumor-related biomarkers	[118]
Bismuth Oxide Nanoparticles, Cisplatin, and Baicalein-Rich Fraction from *O. indicum*	MCF-7, MDA-MB-231, and NIH/3T3	-	The combination demonstrated the most potent radiosensitization effects across all radiation types in cancer cells	[119]
Baicalein and Capecitabine	MCF-7	Thirty-two adult female albino rats	In MCF-7 cells, the combination of compounds enhanced cytotoxicity, cell cycle arrest, and apoptosis compared to individual treatments In rats, baicalein partially mitigated capecitabine-induced cardiotoxicity, oxidative stress, and inflammation	[120]
Baicalin and Doxorubicin	MDA-MB-231	-	Baicalein enhanced the sensitivity of MDA-MB-231 cells to doxorubicin by activating autophagy and mitophagy, down-regulating CDK1, and inhibiting Drp1-mediated mitochondrial fission	[121]
Baicalin/baicalein and Doxorubicin or Docetaxel	MCF-7 and HUVEC-ST	-	Baicalin or baicalein enhanced the cytotoxicity of doxorubicin and docetaxel in MCF-7 cells Both flavonoids increased doxorubicin uptake in MCF-7 cells, induced apoptosis, and caused DNA damage	[122]
Baicalin, Epigallocatechin Gallate (EGCG), and Vincristine	EMT-6/P and EMT-6/V	Female BALB/c mice (4–6 weeks old)	Baicalin and EGCG demonstrate strong anti-proliferative effects, especially in vincristine-resistant cancer cells The combination with vincristine had a synergistic effect in resistant cells Combined therapy significantly reduced tumor size and achieved high cure rates in both models with no liver or kidney toxicity	[123]

**Table 8:** Overview of a nanotechnology-based delivery system for baicalin/baicalein in breast cancer research.

Formula	Cell Lines	Animal Models	The Outcome of the Study	Reference
Baicalein-loaded iron oxide nanoparticles	MDA-MB-231 and HBL-100	-	Baicalein-loaded nanoparticles showed significant cytotoxicity against MDA-MB-231 cells (IC_50_ = 22 µg/mL) Baicalein-loaded nanoparticles disrupted the cell cycle and induced apoptosis by downregulation of the anti-apoptotic protein Bcl-2 and upregulation of pro-apoptotic proteins (Bax, cytochrome-c, caspase-3, PARP, and p53)	[125]
Baicalin-loaded gold nanoparticles (AuNPs) conjugated with thiolated beta-cyclodextrin	MCF-7	-	The obtained delivery system enhanced the apoptotic effects of baicalin	[126]
Baicalein and paclitaxel nanoemulsion	MCF-7 and MCF-7/Tax	Female BALB/c nude mice (6–8 weeks old)	The obtained nanoemulsions enhanced anticancer efficacy through synergistic effects, increased cellular uptake, oxidative stress induction, and apoptosis activation In an MCF-7/Tax xenograft model, nanoemulsion showed the highest tumor inhibition rate (77.0%) and minimal weight loss	[127]
Hyaluronic acid-decorated nanostructured lipid carriers (NLCs) as nanocarriers for co-delivery of baicalein and doxorubicin	MCF-7/ADR	Kunming mice (4–6 weeks old)	The co-delivery system exhibited enhanced targeting, sustained drug release, and synergistic anticancer effects (IC_50_ = 0.056 mg/mL) The obtained formula demonstrated the highest tumor inhibition rate (88%) in mice bearing human breast cancer	[128]
Baicalein-loaded iron oxide nanoparticles	MDA-MB-231	-	Baicalein-loaded iron oxide nanoparticles effectively internalized into MDA-MB-231 cells, localized in critical cellular regions, and induced apoptosis through mitochondrial dysfunction, DNA damage, and cell cycle arrest.	[129]
Baicalin-loaded polylactide-glycolide (PLGA) nanocapsules	MCF-7 and MDA-MB-231	-	Nanocapsules demonstrated superior anticancer activity and induced apoptosis, sustained drug release, and enhanced cellular uptake	[130]
Methoxy poly(ethylene glycol)-folic acid-decorated zeolitic imidazolate framework (ZIF-8) loaded with baicalin	MCF-7 and L929	Female BALB/c mice	The nano-delivery system demonstrated excellent tumor-targeting capability, pH-responsive drug release, and potent anticancer activity both in vitro and in vivo.	[131]
Folate-modified albumin baicalin-loaded nanoparticles	MCF-7	Female BALB/c nude mice (6–8 weeks old)	Nanoparticles demonstrated excellent tumor-targeting capability, sustained drug release, and potent anticancer activity both in vitro and in vivo The obtained system enhanced cellular uptake, induced apoptosis, and significantly inhibited tumor growth	[132]
Baicalein and cinnamon essential oil nanoemulsion	MDA-MB-231	-	Nanoemulsion demonstrated enhanced anticancer activity 19-fold and 23-fold higher cytotoxicity compared to free baicalein after 12 h and 24 h, respectively, against breast cancer cells The anticancer activity is attributed to the combined effect of baicalein and cinnamon oil, along with improved drug permeability and cellular uptake via endocytosis	[133]
Hyaluronic acid-modified ferrous baicalein nanoparticle	HUVECs and 4T1	Female BALB/c mice (8 weeks old)	In 4T1 tumor-bearing mice, nanoparticles and near-infrared significantly inhibited tumor growth (a combination of photothermal therapy and chemotherapy), showed tumor cell necrosis/apoptosis with no damage to major organs	[134]
Folate-modified albumin baicalin-loaded nanoparticles	MCF-7	Female BALB/c nude mice	Nanoparticles demonstrated sustained drug release, enhanced cellular uptake, and potent anti-cancer effects through induction of cell cycle arrest, apoptosis, and autophagy	[135]
Silver nanoparticles obtained by *S. multicaulis* leaf extract	MDA-MB-231 and HFF2	-	The green-synthesized silver nanoparticles demonstrated significant antioxidant, anticancer, and apoptotic activities	[136]

## Data Availability

No new data were created or analyzed in this study. Data sharing is not applicable to this article.

## Supplementary Materials

The following supporting information can be downloaded at https://www.mdpi.com/article/10.3390/cimb47030181/s1.

## Abbreviations

The following abbreviations are used in this manuscript:

4T1	Mouse mammary carcinoma cell line
AKT	Protein Kinase B
ALOX12/15	Arachidonate 12/15-Lipoxygenase
AR	Androgen Receptor
AuNPs	Gold Nanoparticles
Bax	Bcl-2-Associated X Protein
BBB	Blood-Brain Barrier
Bcl-2	B-Cell Lymphoma 2
BCT	Breast-Conserving Therapy
CCID	Cell Cycle Inhibitory Dose
CDK4/6	Cyclin-Dependent Kinase 4/6
COX-2	Cyclooxygenase-2
CSC	Cancer Stem Cells
CYP1	Cytochrome P450 Family 1
CYP1B1	Cytochrome P450 Family 1 Subfamily B Member 1
DDIT4	DNA Damage-Inducible Transcript 4
Drp1	Dynamin-Related Protein 1
E2	Estradiol
EGCG	Epigallocatechin Gallate
EMT	Epithelial-Mesenchymal Transition
ER	Estrogen Receptor
ERK	Extracellular Signal-Regulated Kinase
ERα	Estrogen Receptor Alpha
FAK	Focal Adhesion Kinase
GI Absorption	Gastrointestinal Absorption
GLOBOCAN	Global Cancer Observatory
GPR30	G Protein-Coupled Estrogen Receptor 30
H-Bond	Hydrogen Bond
HCT-116	Human Colon Cancer Cell Line
HER2	Human Epidermal Growth Factor Receptor 2
HIF-1α	Hypoxia-Inducible Factor 1-Alpha
HR+	Hormone Receptor-Positive
HUVECs	Human Umbilical Vein Endothelial Cells
IC_50_	Half-Maximal Inhibitory Concentration
IFIT2	Interferon-Induced Protein with Tetratricopeptide Repeats 2
IGF-I	Insulin-Like Growth Factor 1
IL-10	Interleukin 10
IL-6	Interleukin 6
IORT	Intraoperative Radiotherapy
JNK	c-Jun N-Terminal Kinase
LDH	Lactate Dehydrogenase
Log K_p_	Logarithm of Skin Permeation Coefficient
LogP_o/w_	Logarithm of Octanol-Water Partition Coefficient (lipophilicity measure)
LogS	Logarithm of Aqueous Solubility
LOX-12	Lipoxygenase-12
MAPK	Mitogen-Activated Protein Kinase
MCF-10A	Non-Tumorigenic Breast Epithelial Cell Line
MDA-MB-231	Human Triple-Negative Breast Cancer Cell Line
MDA-MB-468	Human Breast Cancer Cell Line
miRNA	MicroRNA
MMP	Matrix Metalloproteinase
MMTV-PyMT	Mouse Mammary Tumor Virus-Polyoma Middle T Antigen
MOFs	Metal-Organic Frameworks
mTOR	Mammalian Target of Rapamycin
NF-κB	Nuclear Factor Kappa B
NLC	Nanostructured Lipid Carrier
NOD/SCID	Non-Obese Diabetic/Severe Combined Immunodeficiency
P-gp	P-glycoprotein (efflux transporter)
PARP	Poly (ADP-Ribose) Polymerase
PD-L1	Programmed Death-Ligand 1
PI3K	Phosphoinositide 3-Kinase
PIM-1	Proto-oncogene serine/threonine-protein kinase
PLGA	Polylactic-co-Glycolic Acid
PRISMA	Preferred Reporting Items for Systematic Reviews and Meta-Analyses
PROCR	Protein C Receptor
ROS	Reactive Oxygen Species
SATB1	Special AT-Rich Sequence-Binding Protein 1
SGNI	Shuganning Injection
SREBP1	Sterol Regulatory Element-Binding Protein 1
STAT3	Signal Transducer and Activator of Transcription 3
TGF-β1	Transforming Growth Factor Beta 1
TNBC	Triple-Negative Breast Cancer
TNF-α	Tumor Necrosis Factor Alpha
TPSA	Topological Polar Surface Area
u-PA	Urokinase-Type Plasminogen Activator
VEGF	Vascular Endothelial Growth Factor
ZEB1	Zinc Finger E-Box Binding Homeobox 1
ZIF-8	Zeolitic Imidazolate Framework 8
